# Case Report: An Atypical Form of Familial Partial Lipodystrophy Type 2 Due to Mutation in the Rod Domain of Lamin A/C

**DOI:** 10.3389/fendo.2021.675096

**Published:** 2021-04-19

**Authors:** Carolina Cecchetti, M. Rosaria D’Apice, Elena Morini, Giuseppe Novelli, Carmine Pizzi, Uberto Pagotto, Alessandra Gambineri

**Affiliations:** ^1^ Division of Endocrinology and Diabetes Prevention and Care, Department of Medical and Surgical Sciences (DIMEC), Azienda Ospedaliero-Universitaria di Bologna, Bologna, Italy; ^2^ Laboratory of Medical Genetics, Tor Vergata Hospital, Rome, Italy; ^3^ Department of Biomedicine and Prevention, University of Rome “Tor Vergata”, Rome, Italy; ^4^ Unit of Cardiology, Department of Specialistic, Diagnostic and Experimental Medicine, Azienda Ospedaliero-Universitaria di Bologna, Bologna, Italy

**Keywords:** lipodystrophy, rod domain, *LMNA* gene, cardiomyopathy, laminopathy

## Abstract

**Purpose:**

Familial partial lipodystrophy type 2 (FPLD2) patients generally develop a wide variety of severe metabolic complications. However, they are not usually affected by primary cardiomyopathy and conduction system disturbances, although a few cases of FPLD2 and cardiomyopathy have been reported in the literature. These were all due to amino-terminal heterozygous lamin A/C mutations, which are considered as new forms of overlapping syndromes.

**Methods and Results:**

Here we report the identification of a female patient with FPLD2 due to a heterozygous missense variant c.604G>A in the exon 3 of the *LMNA* gene, leading to amino acid substitution (p.Glu202Lys) in the central alpha-helical rod domain of lamin A/C with a high propensity to form coiled-coil dimers. The patient’s cardiac evaluations that followed the genetic diagnosis revealed cardiac rhythm disturbances which were promptly treated pharmacologically.

**Conclusions:**

This report supports the idea that there are “atypical forms” of FPLD2 with cardiomyopathy, especially when a pathogenic variant affects the lamin A/C head or alpha-helical rod domain. It also highlights how increased understanding of the genotype-phenotype correlation could help clinicians to schedule personalized monitoring of the lipodystrophic patient, in order to prevent uncommon but possible devastating manifestations, including arrhythmias and sudden death.

## Introduction

Lipodystrophy syndromes are a group of rare and heterogeneous disorders characterized by variable loss of adipose tissue. The affected patients generally develop a wide variety of severe metabolic complications, especially insulin resistance and hypertriglyceridemia, non-alcoholic fatty liver disease/non-alcoholic steatohepatitis (NAFLD/NASH) and, frequently, uncontrolled diabetes (1, 2). Lipodystrophies can be acquired or congenital, generalized or partial (1, 3). Among the congenital forms, multiple phenotypes have been identified, each with distinct clinical features which can guide diagnostic and monitoring approaches (1, 3). The most frequent form of congenital lipodystrophy is a partial form caused by heterozygous missense pathogenic variants in the *LMNA* gene, which is called familial partial lipodystrophy type 2 (FPLD2: # 151660) or Dunnigan syndrome (4–6).

The *LMNA* gene encodes lamins A and C, ubiquitous filamentous proteins belonging to the type V intermediate filament groups, involved in the correct assembly and structural integrity of the inner nuclear membrane (6, 7). Pathogenic variants can be found throughout the whole Lamin A/C gene, and are known to be the cause of an ensemble of diseases called “laminopathies”. These include FPLD2, cardiomyopathies (dilated cardiomyopathy and conduction system disturbances), myopathies, neuropathies and syndromic forms, classically represented by progeroid syndromes (7–9).

Interestingly, it has been suggested that the *LMNA* genotype and laminopathy phenotype are not randomly associated (10, 11). The proximal C-terminal domain of A-type lamins, coded by exon 8, harbors the majority of FPLD2-linked pathogenic variants, with a specific hotspot at codon 482. This form of FPLD2 is not associated with dilated cardiomyopathy and/or cardiac rhythm disturbances (12). On the other hand, *LMNA*-related cardiomyopathies are generally associated with pathogenic variants involving the alpha-helical rod domain or the head domain of lamins A/C, the former codified by exon 3 and not associated with the lipodystrophic phenotype (13).

The typical phenotype of FPLD2 is the loss of fat in the limbs, trunk, hips and buttocks, and concomitant fat accumulation in the face, neck, axillae, dorsal region, labia majora and visceral region (1–6). The result is a cushingoid appearance, with particular limb muscular hypertrophy and phlebomegaly (1–6). Disease onset is around puberty and is more evident in females than males (6). Over a lifetime and at early age, the affected subjects are at major risk of developing severe metabolic complications and ischemic heart disease in the setting of accelerated atherosclerosis (4, 5).

FPLD2 patients are therefore not usually affected by primary cardiomyopathy and conduction system disturbances, although a few cases of Dunnigan type lipodystrophy and cardiomyopathy have been reported in the literature, all due to amino-terminal heterozygous lamin A/C mutations, which are considered as new forms of overlapping syndromes (14).

Here we report the identification of a female patient with FPLD2 and cardiac rhythm disturbances with a missense mutation affecting the lamin A/C rod domain who referred to our unit with suspicion of Cushing syndrome.

## Materials and Methods

### Physical Examination and Biochemical Assays

Physical examination included anthropometry (height, weight, waist and hip circumferences), blood pressure, estimated signs of hypercortisolism and hyperandrogenism, and evaluation of acanthosis nigricans. Height was measured without shoes and rounded to the nearest 0.5 cm; weight was measured without clothes; Body Mass Index (BMI) was calculated as weight (kg) divided by the square of height (m). Waist circumference was measured with the subject standing, with a 1 cm wide measuring tape, in accordance with WHO guidelines (15). Blood pressure was measured twice in the supine position, in the morning, after at least 3 min of rest before measurements, taking the average of two.

Blood sample measurements included hematology, metabolic parameters and hormones. An oral glucose tolerance test (OGTT) with 75 g of glucose (Curvosio, Sclavo, Cinisello Balsamo, Italy) and a 1 mg overnight dexamethasone (Decadron; Visufarma, Rome, Italy) suppression test (DST) was also performed.

Blood samples were taken and all tests were carried out in the morning after 12-h overnight fasting. Glucose, insulin, triglycerides, total and high-density lipoprotein (HDL) cholesterol, LH, FSH, estradiol and testosterone were measured by Modular Analytics E170 (Roche Diagnostics, Mannheim, Germany), cortisol was measured by electrochemiluminescence immunometric assay (Elecsys E170; Roche Diagnostics, Indianapolis, IN, USA), and ACTH by chemiluminescent immunoenzymatic assay (Immulite2000; Siemens Healthcare Diagnostics, Tarrytown, NY, USA) as reported elsewhere (16, 17). Low-density lipoprotein (LDL) cholesterol was calculated using the Friedewald formula (18). Insulin resistance was calculated using the homeostatic model assessment of insulin resistance (HOMA-IR) index (19).

### Dual-Energy X-Ray Absorptiometry

To determine body composition (whole body and regional fat in the head, trunk, upper and lower extremities), dual-energy X-ray absorptiometry (DXA) was performed using a Lunar iDXA densitometer (DXA; GE Lunar iDXA, GE Healthcare, Bucks, UK).

### Cardiac MR Images, 24-h Continuous ECG Monitoring, Exercise Stress Test, Echocardiography and Coronary CT Angiography

Cardiac MR images were obtained using a 1.5 Tesla imaging machine (Signa Twin Speed Excite, General Electric, Milwaukee, Wisconsin, USA). Cardiac MR protocol included multiplanar cine imaging, short-tau inversion recovery (STIR), late gadolinium enhanced imaging and native T1 and T2 mapping (20).

Twenty-four-hour electrocardiographic monitoring for heart rate variability analysis was also performed. Analysis of electrocardiographic tapes was performed using Del Mar Avionics Accuplus 363 in confirm-option, which enables manual labelling of each artefact, premature beat, pause and any changes in the cardiac rhythm.

The patient also underwent a symptom/sign-limited Bruce treadmill exercise stress test (EST) under continuous ECG monitoring. The EST was considered positive for myocardial ischemia when a horizontal or downsloping ST-segment depression ≥ 1 mm or an upsloping ST-segment depression ≥ 1.5 mm at 0.08 s from the J point was detected. Criteria for EST interruption included physical exhaustion, worsening symptoms (angina, dyspnea), occurrence of any potentially dangerous clinical condition (e.g., pre-syncope, hypotensive or hypertensive response, arrhythmias), ST-segment elevation ≥ 1 mm or ST-segment depression ≥ 3 mm in two or more contiguous leads.

The patient also underwent transthoracic echocardiography (TTE) that was performed by using Philips EPIQ ultrasonography machines. Left ventricular ejection fraction was calculated with the biplane Simpson**’**s method acquiring volumes in both 4- and 2-chamber views, according to the European Association of Cardiovascular Imaging Guidelines (21).

Computed tomography coronary angiography (CTCA) was finally planned to rule out coronary artery disease. The examination was carried out by a 128-slice computed tomography (Revolution GSI, GE Healthcare) with 0.35 second rotation time, prospective ECG triggering and 40 mm total collimation width. The angiographic scan was preceded by a baseline acquisition for calcium score evaluation. Therefore, 100 ml of high concentration contrast medium (95 ml of Iomeron 400, Bracco, Italy) were injected at 5 ml/s and bolus tracking technique was used to synchronize the start of the acquisition with contrast bolus arrival. Images were reconstructed using the GE advantage workstation 4.6 (GE Healthcare).

### Genotyping

DNA sequencing analysis was performed using a custom-built Next-Generation Sequencing (NGS) panel which includes the main genes associated with lipodystrophy (*LMNA* NM_170707*, PPARG* NM_015869*, PLIN1* NM_001145311*, CIDEC* NM_001199551, *LIPE* NM_005357*, AKT2* NM_001626*, CAV1* NM_001753) (2). Ion AmpliSeq™ Designer tool (https://www.ampliseq.com/) was used to design a specific primer pool to amplify the entire coding sequence (CDS) plus 25bp at the exon-intron junction of each gene. Two different primer pools were obtained, for a total of 114 amplicons. The calculated coverage of the coding sequence with a minimum depth of coverage of 50× is about 90.2%, with an exon padding of 25 bp. Regions not completely covered by NGS design or covering less than 50x were analyzed by the Sanger sequencing method (primers available on request).

NGS analysis was performed on genomic DNA (gDNA) extract from a peripheral whole blood sample (200µl) using a Biorobot EZ1 automated system and the EZ1 DNA Blood Kit (Qiagen, Hilden, Germany). DNA quality and concentration were checked by Nanodrop (NanoDrop^®^ ND-1000, Euroclone). Ten nanograms of gDNA from each patient were subjected to a multiplex amplification using Ion AmpliSeq™ Library Kit (Thermo Fisher Scientific, Waltham, Massachusetts, USA) according to the manufacturer’s instructions. After amplification, the two pools were then mixed together for the template preparation and for the sequencing analysis using Ion 510™ & Ion 520™ & Ion 530™ Kit – Chef (Thermo Fisher Scientific, Waltham, Massachusetts, USA) on Ion Torrent S5 platform (Thermo Fisher Scientific, Waltham, Massachusetts, USA).

Data obtained from sequencing were processed with Torrent Suite Software (Thermo Fisher Scientific, Waltham, Massachusetts, USA), which generates filtered sequences after quality control of the reads and summarizes different information on the run such as the uniformity, coverage analysis and variants found (Variant Caller). All the sequences were aligned with the Human Reference Genome assembly (GRCh37/hg19). Data analysis produces a complete report that includes the following files: FASTQ (base calls of all the reads produced and the quality score of each base); BAM (alignment of the reads over the reference genome); Variant Call File (VCF) (the chromosomal position, name and reference genome of each variant).

The variants were identified using Integrative Genomics Viewer software (IGV, Broad Institute) (http://www.broadinstitute.org/igv/) and all the identified variants (splice, stop, synonymous, non-synonymous, insertion, or deletion) were annotated with the ANNOVAR tool or using online Ion Reporter software (https://ionreporter.lifetechnologies.com/ir/secure/home.html).

The variants identified were searched using the following databases: Human Gene Mutation Database (HGMD; http://www.hgmd.cf.ac.uk), UMD (http://www.umd.be/), gnomAD v2.1.1 (http://gnomad.broadinstitute.org/), ClinVar (https://www.ncbi.nlm.nih.gov/clinvar/). Classification was confirmed with the help of VarSome software (https://varsome.com/).

Sanger sequencing, using ABI 3130 XL Genetic Analyzer (Life Technologies), was used to confirm the candidate variants. Primer pairs used to amplify fragments encompassing individual variants were designed using the online tool Primer3Plus (http://www.bioinformatics.nl/cgi-bin/primer3plus/primer3plus.cgi) (sequences available on request), and PCR amplifications were performed using 50 ng of gDNA following the AmpliTaq Gold^®^ DNA Polymerase protocol (Applied Biosystem, Thermo Fisher Scientific Waltham, Massachusetts, USA).

A written consent was obtained from the patient to publish the case in anonymous form.

## Results

A 34-year-old untreated woman was referred to our unit with suspicion of Cushing syndrome. She had recently developed myalgias, severe asthenia, sleep disturbance and depression.

The patient also complained of lower extremity swelling that had already been evaluated with doppler ultrasonography which was pathologically negative.

No significant events were reported in the past; menarche occurred at 13 years of age, with subsequent oligo-amenorrhea ever since with an ovarian morphological aspect compatible with polycystic ovary syndrome. At 24 years she had an uncomplicated pregnancy.

**Figure 1:**
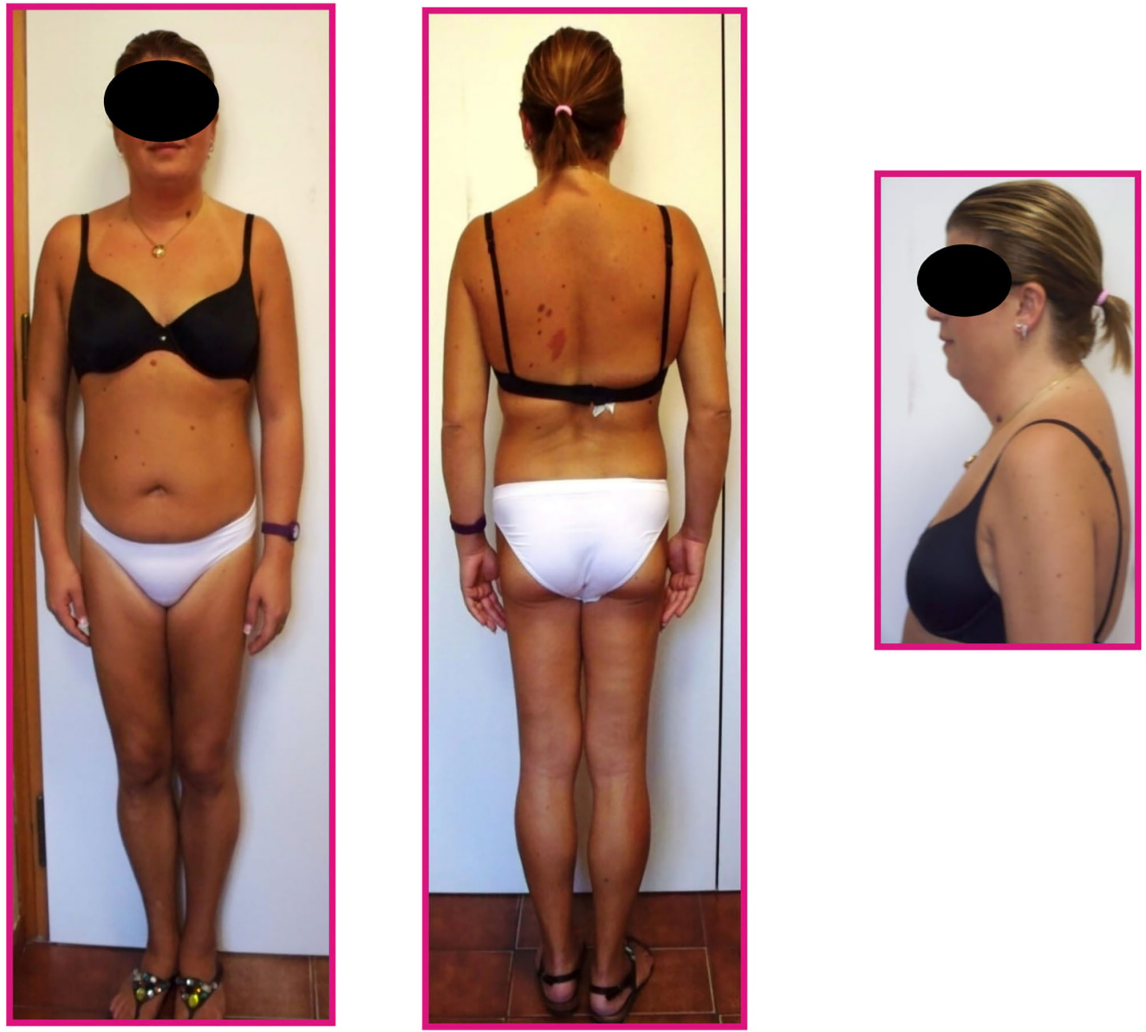
**(A)** DNA analysis sequencing of the proband by NGS and Sanger sequencing revealed the heterozygous c.604G>A change in *LMNA* gene. **(B)** Lamin A and location of probable pathogenic variant identified in our patient. **(C)** Alignment of the human lamin A aminoacidic sequence with homologous proteins. The affected amino acid is highlighted in black.

Physical evaluation revealed an altered adipose tissue distribution suggestive of Dunnigan-type lipodystrophy; she had atrophy of subcutaneous fat in the lower limbs and normal fat representation in the trunk and arms, in spite of increased accumulation around the neck, face and dorsocervical region (**Figure 1**). Muscular pseudohypertrophy and phlebomegaly (prominent veins) were also visible in the lower extremities. The patient reported that the particular phenotype started to be noticeable around puberty.

There was no evident hirsutism. BMI was 25.4 kg/m^2^, with a waist circumference of 86 cm.

Blood pressure and heart rate were within normal range. At abdominal evaluation, the liver was palpable with regular margins. No other significant anomalies were found. Abdominal ultrasound was performed which revealed increased liver echogenicity related to mild steatosis.

The patient was an only child. Her father reportedly had features of lipodystrophy and died at 54 years of age due to congestive heart failure. Her mother was 72-year-old, without a lipodystrophic phenotype, diabetes, or cardiovascular diseases. No information on grandparents was available. The daughter recently gave consent to perform genotyping (results still ongoing).

Initial hormonal evaluation showed normal levels of ACTH and cortisol, with an adequate response to DST (8.00 am cortisol 0.29 µg/dl), thus excluding a diagnosis of Cushing syndrome. Basal LH levels were slightly elevated, with unaltered FSH and estradiol levels (**Table 1**). Lipid profile was overall adequate, with the exception of slightly elevated levels of LDL cholesterol (**Table 1**). Serum aspartate transaminase (AST) and alanine aminotransferase (ALT) were not altered.

**Table 1:** Clinical, hormonal and biochemical profile of the patient at baseline.

Parameters		*Local reference values*
***Pharmacotherapy***	none	
***Clinical data:***		
Age (years)	34	
BMI (kg/m^2^)	25.4	
Waist circumference (cm)	86	
WHR	0.95	
Systolic blood pressure (mmHg)	130	
Diastolic blood pressure (mmHg)	80	
***Total Body DXA:***		
Fat mass, total (%)	30.2	≥29
Fat mass, lower extremities (%)	23.2	n.a.
Fat mass, higher extremities (%)	32.6	n.a.
Fat mass, trunk (%)	35.2	n.a.
Fat Mass Ratio	1.49	<1.2
***Biochemistry:***		
LH (mU/ml)	10.3	2.1-10.9
FSH (mU/ml)	7.4	3.9-8.8
Estradiol (pg/ml)	38	22-115
Glucose, fasting (mg/dl)	74	60-100
Insulin, fasting (µU/ml)	20	<10
Glucose, 120min OGTT (mg/dl)	108	<140
Insulin, 120min OGTT (µU/ml)	357	<60
HOMA-IR	3.65	≤2.5
HbA1c (mmol/ml)	33	20-42
Total cholesterol (mg/dl)	200	<200
HDL-cholesterol (mg/dl)	45	>45
Triglycerides (mg/dl)	110	<150
LDL-cholesterol (mg/dl)	131	<100
CPK (UI/l)	70	60-190

BMI, body mass index; WHR, waist-to-hip ratio; HOMA-IR, homeostasis model assessment-insulin resistance index; CPK, creatine phosphokinase; DXA, dual-energy X-ray absorptiometry; Fat Mass Ratio = % of the trunk fat mass/% of the lower extremities fat mass (n.v. < 1.2; ref. 34); OGTT, oral glucose tolerance test. n.a., not available.

On the other hand, severe fasting hyperinsulinemia and a condition of insulin resistance (HOMA-IR of 3.65) were found (**Table 1**). The OGTT revealed normal glucose tolerance but notably high levels of glucose-stimulated insulin, thus confirming the severe insulin resistance. The glycated hemoglobin (HbA1c) level was normal (**Table 1**). Since the patient complained of myalgia in the lower extremities, creatine phosphokinase levels were evaluated which were within the normal range (**Table 1**). Metformin therapy at the dose of 1700 mg per day and omega-3 polyunsaturated fatty acids (PUFA) at the dose of 1 gr per day were started.

**Figure 2:**
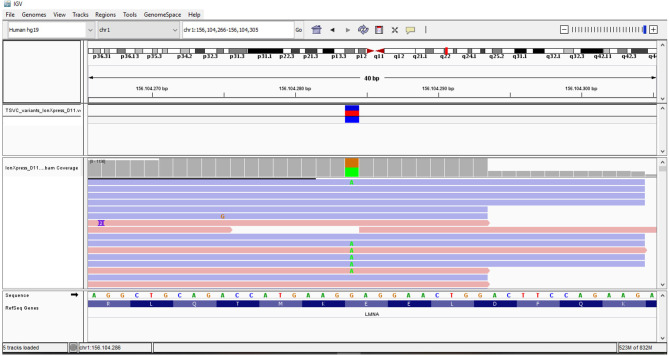
Body fat distribution pattern of the patient.

The association of the particular phenotype and the severe insulin resistance state led to the suspicion of a lipodystrophic syndrome. Total body DXA was performed and the results were suggestive of partial lipodystrophy (**Table 1**) (35). Accordingly, NGS analysis of the genes associated with familiar partial lipodystrophies were carried out. These revealed four exonic variants in three of the seven genes analyzed (**Table 2**). A heterozygous missense variant c.604G>A was found in the exon 3 of the *LMNA* gene, leading to amino acid substitution glutamic acid to lysine (p.Glu202Lys) (**Figure 2**). This variant was absent from the controls in the GnomAD database and was reported in a recent paper in a patient with neuromuscular disease (22). Computational analysis using Varsome software (https://varsome.com/) classified the p.Glu202Lys variant as probably pathogenic due to its location in a mutational hot spot, and bioinformatics analyses supported its deleterious effect. The variant is located in the central alpha-helical rod domain of lamins A/C with a high propensity to form coiled-coil dimers. Given that a missense pathogenetic variant in the close amino acid, c.607G>A p.Glu203Lys, has been identified in patients with familial dilated cardiomyopathy and conduction system disease, the geneticist advised us to rule out possible cardiac anomalies (23). In the meantime, with the suspicion of obstructive sleep apnea syndrome (OSAS), polysomnography was performed. The results came back negative for any sleep disturbances. However, highly frequent ectopic ventricular beats were detected on normal resting 12-lead ECG and on ECG monitoring during polysomnography, which were often grouped in bigeminy and trigeminy. The abnormal ECG findings were then confirmed by 24-h holter ECG, which demonstrated frequent monomorphic premature ventricular complexes in bigeminy and rare premature supraventricular beats. To clarify the newly discovered anomalies, further cardiac investigations were required. A TTE and a cardiac MRI were executed which showed no significant abnormalities; left and right ventricle had normal diameter and contractility, no valvular alteration and pericardial disease were observed and there was no evidence of cardiac fibrosis. EST showed unclear results for inducible ischemia, thus requiring CTCA with no evidence of atherosclerotic lesions. Other causes of cardiac rhythm disturbances (e.g., electrolyte alterations, thyroid disease, use of pro-arrhythmic substances) were excluded. The 24-holter ECG monitoring was repeated 6 months later which showed progressively worsening of aberrant ventricular beats and repolarization anomalies; and the patient was thus started on beta-blockers (metoprolol at daily dose of 75mg). Six months after starting the beta-blocker therapy, the aberrant ventricular beats and repolarization anomalies disappeared, as did the reported sleep disturbances which we had previously suspected to be OSAS. The patient is regularly followed-up in our unit with biochemical analyses every 6 months, cardiological evaluation with 24-holter ECG monitoring plus TTE every 12 months, and annual abdominal ultrasonography. A total body DXA is performed every 24 months to assess the evolution of lipodystrophy.

**Table 2:** Genetic variants identified after NGS followed by adequate filtering.

Position	Genotype	Gene	Transcript	Function	Exon	Protein	Coding	dbsnp	Clinvar	Varsome
chr1:156104284	G/A	LMNA	NM_170707.3	missense	3	p.Glu202Lys	c.604G>A			Likely Pathogenic
chr1:156107534	C/T	LMNA	NM_170707.3	synonymous	10	p.(=)	c.1698C>T	rs4641	Benign	
chr9:139571430	G/A	AGPAT2	NM_006412.3	missense	3	p.Arg159Cys	c.475C>T	rs142993240	Benign	
chr15:90213229	C/C	PLIN1	NM_001145311.1	missense	5	p.Pro194Ala	c.580C>G	rs6496589	Likely Benign	

## Discussion

This report provides further evidence of the possible association of Dunnigan type lipodystrophy with cardiomyopathy in pathogenic variants of the *LMNA* gene, in this case affecting the protein alpha-helical rod domain.

Laminopathies are a heterogenous group of inherited disorders caused by pathogenic variants of the *LMNA* gene located on chromosome 1q21, which encodes nuclear lamins A and C (8).

Lamins are thought to play an important structural and regulatory role; first they polymerize to form the nuclear lamina, a filamentous network involved in maintaining the shape of the nucleus. In addition, they interact with integral proteins of the inner nuclear membrane and chromatin itself, regulating various transcriptional events downstream of diverse signaling pathways. Lamins therefore represent a key element in physiological cell processes (24, 25). Lamins are type V intermediate filaments and are structured in a central alpha helical coiled cod domain, flanked by a large C-terminal tail and a shorter N-terminal head (25).

The alpha-helical domain, responsible for the dimer formation of lamins A and C, is coded partially by exon 1 of the *LMNA* gene, which also yields the head domain, and exons 2-6; the rod domain is highly structured and spans almost half of the entire protein. On the other hand, exons 6-9 encode for the C-terminal tail, including the portion of lamins that interact directly with DNA or regulatory proteins and the region of nuclear localization signal (NLS), which tether lamins to the nucleus (26).

FPLD2 is the most frequent form of congenital lipodystrophy which is commonly associated with pathogenic variants that affect the C-terminal domain of A-type lamins (12, 27, 28).

Although a clear correlation between clinical manifestations and genetic alterations has not been established, a non-random genotype-phenotype correlation might exist in laminopathies (10).

*LMNA* pathogenic variants located upstream of the NLS tend to manifest with disturbances in the skeletal and cardiac systems, whereas metabolism and bone alterations, together with progeroid phenotypes, tend to be associated with pathogenic variants occurring downstream of the NLS (26).

The central rod-domain coding regions are in fact upstream of the NLS. Several pathogenic variants affecting the lamin A/C rod-domain have been linked to cardiac impairment, in the form of conduction system disturbances and dilated cardiomyopathy, which are typical features of *LMNA*-linked cardiac disturbances (9, 13).

The mechanisms of cardiac pathogenicity are unknown. Some hypotheses have been suggested, but more studies are needed (29). Hypothetically, alterations affecting the alpha-helical rod domain may cause cardiac disturbances by impairing interactions with particular cytoplasmic proteins, for example filamentous proteins such as desmin or the actin-based cytoskeleton, which are known to malfunction in dilated cardiomyopathies linked to different genes (13).

In reality, lamin A/C alterations causing cardiomyopathy have been found throughout the *LMNA* gene. Of note, *LMNA* is one of the most commonly affected genes in patients with dilated cardiomyopathy.

In dilated cardiomyopathy due to lamin A/C mutation (DCM1A), signs of lipodystrophy are usually uncommon. On the other hand, primary cardiac impairment is rare in patients with FPLD2 caused by exon 8 pathogenic variants, in which cardiovascular events are usually linked to atherosclerotic vascular disease accelerated by severe metabolic disturbances (24, 25, 30).

Interestingly, sporadic cases of Dunnigan-type lipodystrophy with primary cardiomyopathy have already been described in the literature (31). Subramanyam et al. (14) reported a series of laminopathic patients showing primary cardiomyopathy in association with the typical partial lipodystrophic phenotype, in which pathogenic variants were curiously found on the head and rod domain. The fat loss pattern and metabolic alterations in these patients did not differ from those observed in exon-8 mutated FPLD2 individuals. These subjects also suffered from cardiac impairment as seen in *LMNA*-associated dilated cardiomyopathy without lipodystrophy. The alterations ranged from rhythm disturbances, such as persistent atrial fibrillation and/or various grades of heart block, and idiopathic dilated cardiomyopathy with heart failure, generally arising at a young age.

More recently, a large scale, single-center study compared patients carrying R482 *LMNA* pathogenic variants with non-R482 mutated subjects (28). The results confirmed a low frequency of arrhythmias and dilated cardiomyopathy in patients carrying “typically lipodystrophic” R482 *LMNA* pathogenic variants, with an expected higher incidence of coronary atherosclerosis. On the other hand, patients with non-R482 pathogenic variants often showed a worse cardiac phenotype, including an initial conduction or rhythm disorder associated with a non-negligible risk of sudden death (28). Cellular studies confirm that FPLD2 functional syncytia of mature human induced pluripotent stem cell-derived cardiomyocytes (hiPSC-CMs) presented several rhythm alterations such as early after depolarizations, spontaneous quiescence and spontaneous tachyarrhythmia (32).

To summarize, in a young lipodystrophic patient, we found a rare *LMNA* mutation located in exon 3, which affected the protein alpha-helical rod domain. The patient showed typical signs of FPLD2 associated with mild metabolic disturbances and initial cardiac impairment, in the form of multiple ventricular ectopic beats and repolarization anomalies.

We believe that although the cardiac disturbances are mild in this case, careful monitoring of the patient is essential. In DCM1A, ECG abnormalities are usually one of the first signs of disease presentation and progression (33). In fact, minor ECG changes including premature ventricular contractions (together with conduction system disease and/or arrhythmias) commonly precede the development of left ventricular dysfunction, when it is not present at the time of diagnosis. Of note, in laminopathic patients affected by muscular dystrophies or asymptomatic at the time of genetic diagnosis, cardiac disturbances can also manifest many years later. Moreover, sudden death, occurring even in asymptomatic patients, has been linked to *LMNA* pathogenic variants. Careful follow-up is thus needed in all laminopathic patients to detect early signs of left ventricular dysfunction and to prevent severe arrhythmic complications, by medical therapy and ultimately by inserting an implantable cardioverter defibrillator.

It is worth noting that our patient was recently evaluated with a 24-h holter ECG, which showed complete recovery of the rhythm disturbances after the patient was started on beta-blockers.

In conclusion, this report supports the idea that there are likely “atypical forms” of FPLD2, especially those where pathogenic variants are not located in *LMNA* exon 8 and the classical lipodystrophic presentation is associated with uncommon disturbances, particularly primary cardiological involvement.

This report also emphasizes how increased understanding of the genotype-phenotype association could help clinicians to schedule personalized monitoring of the lipodystrophic patient, aimed at preventing uncommon but life-threatening manifestations, including arrhythmias and sudden death. At last, an in deep understanding of the genotype-phenotype association could be useful in tailoring the best treatment option. In these atypical forms of FPLD2, cardiological medical therapy might represent a helpful treatment option alongside the therapies already used to manage the metabolic complications. These are the more commonly used therapies such as insulin-sensitizers, antidiabetic or lipid lowering drugs, or, the less commonly used but extremely useful metreleptin therapy, that has proven to be effective in treating major metabolic complications in FPLD2 patients not responding to first line drugs (34).

## Ethics Statement

Written informed consent was obtained from the individual(s) for the publication of any potentially identifiable images or data included in this article.

## Author Contributions

CC, CP, and AG collected clinical data and wrote the manuscript. MD and EM performed the genetical analysis and revised the manuscript. GN and UP revised the manuscript. All authors contributed to the article and approved the submitted version.

## Conflict of Interest

The authors declare that the research was conducted in the absence of any commercial or financial relationships that could be construed as a potential conflict of interest.

The reviewer VG declared a shared affiliation with several of the authors, MA, EM, GN, to the handling editor at time of review.
